# The RNA-First Fallacy: Conflating Evolutionary Ancestry with Prebiotic Primacy

**DOI:** 10.3390/life16050837

**Published:** 2026-05-19

**Authors:** Amit Kahana

**Affiliations:** 1 Institute for Molecules and Materials, Radboud University, 6500 HB Nijmegen, The Netherlands; amit.kahana@ru.nl; 2 School of Chemistry, University of Glasgow, Glasgow G12 8QQ, UK

**Keywords:** RNA world, RNA-first, protobiology, origin of life, abiogenesis, autocatalytic sets, evolution, prebiotic chemistry

## Abstract

The RNA-World hypothesis remains the most widely accepted framework in origins-of-life research, anchored in compelling biochemical evidence for RNA’s deep evolutionary ancestry. However, this viewpoint routinely extends beyond that and is frequently conflated with claims that RNA served as life’s primal substrate. This essay argues that the RNA-First paradigm, in its pursuit of this claim, systematically projects biological biases onto a chaotic and combinatorially vast abiotic landscape. It relies on privileged, highly complex molecular constructs whose spontaneous emergence in such combinatorial settings is overwhelmingly implausible. Critically, the experimental evidence accumulated in support of RNA-First has largely demonstrated the compatibility of RNA with prebiotic conditions, but not its probability, necessity, or chemical precedence over the numerous alternatives that abiotic chemistry affords. The eventual emergence of RNA chemistry demands a preceding protobiological stage, characterized by chemically diverse, collectively autocatalytic molecular networks. Embracing this broader protobiological framework, and confronting the true combinatorial complexity of abiotic chemistry, is essential for a rigorous and unbiased account of life’s origin.

## 1. Conflating Ancestry with Primacy

While the origin of life remains an unsolved mystery, both on the early earth and as a more universal phenomenon, the most widely invoked scenario by researchers to explain it is the RNA-World. The general notion of the RNA-World is that at some point during the first stages of life’s evolution, RNA was a dominant molecular feature of such life forms, preceding the advent of proteins and DNA by fulfilling both a catalytic and a genetic role. The main evidence for this is anchored in modern-day biology, where RNA constructs perform fundamental roles that are shown to be substantially ancient. Indeed, even before the term had been coined by Gilbert [1], its central argument was purely that RNA evolved earlier than proteins and DNA, primarily supported by new evidence at the time that naturally occurring RNA, an essential “informational” polymer, could perform catalytic reactions. Since then, many more publications have reported diverse catalytic capacities of biological and nonbiological RNA sequences [2,3,4,5], including generating peptide bonds at the core of the ribosome [6], giving credence to this early evolution scenario [7].

Alongside the maturation of the RNA-World was the speculation that life may have originated in RNA as a primal or even exclusive substrate (RNA-First) [1,8]. The accumulation of evidence for RNA catalytic capacities bolstered scientists to advocate not only for RNA ancestry but for RNA primacy as well, to the point that the term RNA-World has come to represent both aspects. And while the evidence for RNA ancestry is strong and accepted, supporting an RNA-World evolutionary stage, arguments for RNA primacy are much more tenuous [9]. Nonetheless, significant progress has been made in the investigation of RNA emergence from prebiotic chemistry [10,11] and its evolution within an RNA-dominated molecular environment [12,13], all to justify an RNA-First origins scenario rather than contending with the later emergence of RNA chemistry during evolution [7]. This is perhaps best evidenced in the recent reports of ribozymes with RNA polymerase capabilities, the cornerstone of any RNA-First explanation of life’s origin [3,14,15].

**Figure 1:**
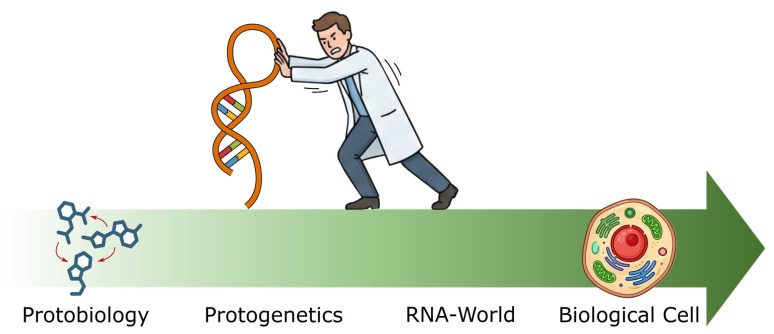
The contentious role of RNA in early evolution. The arrow represents the evolutionary trajectory from protobiology to the biological cell, with Protogenetics and the RNA-World as critical intermediary stages. The RNA-First paradigm implicitly assumes not only that RNA was fundamental to the emergence of genetic material but that the protobiological stage itself was already dominated by RNA chemistry, which this essay argues is incompatible with the combinatorial vastness of abiotic environments.

This prevailing conflation between RNA-World and RNA-First has not seen as much rigorous debate as required for a theory that attempts to explain the origin of life [9]. Modern RNA-First research, promoting RNA as the sole or dominant substrate of life, depicts its motivation as finding solutions to shortcomings in the hypothesis [3,7,16], akin to an engineering task, rather than investigating prebiotic chemistry objectively and discovering supportive evidence for RNA primacy [9]. There are fundamental reasons to believe that the first protocells exhibited vastly different chemistry that was not dominated by ribonucleic acids, or their related species, due to the chaotic nature of abiotic environments [17,18,19,20] and that RNA centrality has emerged later in evolution. The evidence that appeared in many RNA-First reports merely raise the possibility that RNA is compatible with such messy abiotic environments, not that RNA is the essential primal substrate of life (Figure 1).

## 2. Prebiotic Implausibility of RNA-First

It is intuitive to assume, perhaps justly, that RNA-First is a parsimonious hypothesis, as it does not invoke any additional chemical compounds to explain life’s origin. This argument underappreciates the messiness and vastness of abiotic chemistry. Chemical space is enormously large [21], and abiotic environments explore it exhaustively and combinatorially [17,18,19,20]. In such a rich molecular arena, even with a conceivable synthetic preference under some conditions for nucleotide chemistry, the possibility of pure RNA abiogenesis is overwhelmingly slim. In addition, RNA-First substantially relies on the prevalent notion of information as attributed solely to character strings, manifested as encoded polymers in biological cells. This viewpoint easily resonates with biologists and computationally oriented scientists, leading to its popularity within the scientific community. This, again, instills a misunderstanding of abiotic chemistry, where information is initially manifested compositionally and requires much more selective machinery to facilitate meaningful sequence-centric encoding of any kind [9,22].

Nonetheless, significant effort has been made to elucidate the prebiotic origins of the RNA-World, aided by the immense collective experience of analyzing RNA molecules in biological contexts. Key publications present viable prebiotic pathways that produce RNA precursors [10,11,16]; however these pathways are either artificially constrained and engineered to selectively produce predetermined products or are more lenient and therefore generate a rich landscape of chemical compounds. It appears that while RNA synthesis is permitted within prebiotic conditions, there is not necessarily any chemical basis, in the absence of an evolved selection mechanism, that would significantly favor such particular chemistry over any alternatives and avoid a combinatorial explosion.

To tackle this reality, several publications proposed geological and physical prebiotically acceptable methods to concentrate and enrich biomolecules with emphasis on RNA precursors, most prominently through gas bubbles [23], thermal gradients [24], mineral surfaces [25], and membranes [26]. These indeed generate more concentrated and differentiated settings for possible directed reactivity, absolutely useful in explaining early molecular evolution, but they do not provide any intrinsic preference for RNA chemistry or biomolecules at large. They still produce significantly intricate mixtures of molecules based on vaguely similar properties, and even if there was a conceivable sequestration bias towards biochemistry, generating localities dominated by nucleotide chemistry requires implausibly high selectivity. Enrichment strategies thus raise the slight possibility of concentrating RNA building blocks without providing any evidence for the probability this might occur within a rich prebiotic setting.

The sequence space of RNA is a vast combinatorial space, even when limited to four canonical nucleotides. Experimentation limited to such extents is already a notably challenging task. But it pales in comparison with the larger chemical space that is prebiotically accessible. Moreover, even its rudimentary nucleotide building blocks are already more complex than any molecule ever detected in abundance within messy abiotic environments [27], despite conceivable synthetic biases. Therefore, many of the Darwinian properties of RNA-based systems, whether involving a singular functional ribozyme [3] or a catalytic network of RNAs [12,13,28], cannot be deemed entirely emergent or unique. They are dependent on an acquired knowledge of particular manifolds in chemical space that were explored throughout biological evolutionary history and cannot by any means rule out alternative systems displaying similar (or even improved) properties at such evoked molecular complexity. While these systems are enormously informative of network dynamics and general molecular evolution, they are biologically biased, often performed with pure RNA substrates and with seeded complex constructs, and do not contend with combinatorial expansion of the molecular landscape associated with prebiotic chemistry.

## 3. Principles of Protobiology

The essence of the RNA-centric explanation for abiogenesis is the ability of RNA to self-replicate, which has been described in detail through recent discoveries of privileged polymerase ribozymes [3,14]. As argued before, these are complex constructs that are incredibly unlikely to have spontaneously formed in abiotic settings. Simpler RNA constructs that exhibit related functionalities, such as cleavage, ligation, and recombination, are more discoverable yet still significantly too complex for stochastic abiotic formation [29,30]. The reported polymerases are also very nonspecific, leading to a likely dilution of all attained constructs (including the polymerase itself) without any additional regulation elements in the system akin to modern cellular control. Lastly, these ribozymes present their polymerase capabilities in a very limited set of conditions, such as pure oligomeric nucleotide substrates and templates, relying on a strong pre-evolved selective system to carefully maintain them. If the cornerstone of RNA-First relies on a preceding evolved molecular system, then life cannot have originated directly in RNA, rendering the requirement for the replication of RNA constructs purely by ribozyme action as unnecessary. This is arguably true for any chemically constrained origin scenario, such as short, biologically relevant peptides [31]. Even RNA-centric systems that are more permissive, yet constrain their molecular repertoire to few chemical families, are unlikely to constitute a realistic primal model for life’s origin. The emergence of genetic material is therefore exceedingly more plausible to occur through a simpler and more chemically diverse, dynamically evolving catalytic molecular system, which is the essence of protobiology.

In parallel to the development of the RNA-World concept, and partly inspired by it, emerged the investigation into sets of molecules that collectively reproduce, often referred to as autocatalytic sets [32,33]. While it was originally developed for polymers akin to proteins and RNA, it has expanded to include general catalytic networks of molecules, displaying Darwinian properties such as variation and heritability, with real evidence of ancestry within biological metabolic networks [34]. Through mutual catalysis, the molecular sets maintain their composition and facilitate a more directed exploration of chemical space. Notable examples of physically realizable autocatalytic sets are lipid assemblies such as micelles and vesicles and, to a lesser extent, coacervate droplets. These assemblies display substantial catalytic capacities, selectively incorporating molecules through covalent and noncovalent interactions, and thus naturally enriching compounds out of the vast combinatorial space [22]. They also present evolutionary dynamics through growth and division cycles with potentially preserved molecular compositions across generations due to mutual catalytic interactions between constituent molecules, supported by preliminary experimental evidence [22,35,36,37]. Lipid assemblies exhibit notable chemical leniency, befitting an autocatalytic set that does not inherently demand any specific molecular identity or notable seeded complexity. This makes their emergence not merely possible, but statistically far more probable than any consequential ribozyme in a prebiotic context.

Though experimental evidence for collectively autocatalytic sets in small molecules is still scarce [22,28,36], significant validation for this concept in more complex molecules comes from experimental networks of RNAs themselves, cooperatively maintaining network composition over generations through mutual catalysis [12,13,38]. While chemically biased, as described above, they convey the possibility that these molecular networks are not inherently RNA-specific and may arise with aid from physicochemical constraints such as lipid assemblies or mineral surfaces. By expanding the notion of autocatalysis to molecular networks with no strict molecular identities, autocatalytic sets become a substantially more viable evolutionary path from messy abiotic chemistry to cellular life.

Despite the implausibility of the RNA-First scenario, research into the prebiotic origin of ribonucleic acids is helpful to determine how a protobiological molecular system eventually gave rise to RNA-based genetic material. This body of work attempts to ascertain the evolutionary advantages of RNA or nucleotide chemistry in complex chemical settings, such as evidence for tight coevolution with protopeptides [39,40,41], metabolic benefit of nucleotide-like catalytic cofactors [42] and their energetic affordances [43,44]. While these are valuable reports, they are limited in their capacity to determine RNA’s eventual fixation in early evolution, since they operate in combinatorially limited settings, focused on their biologically relevant investigative targets. The discovered advantageous prebiotic properties of RNA, such as its beneficial interactions with peptides, minerals and membranes [25,26,39], have not been determined for many other chemical classes (if at all). The road forward must contend with the vast landscape of abiotic chemical possibilities, extending these research ideas and providing empirical evidence as to what processes gave rise to the RNA-World in much richer contexts and out of pre-existing evolving molecular systems [45]. Research into the advent of RNA chemistry within this context would elucidate the transition from pure compositional inheritance of early evolving molecular networks to heredity comprising more sequential information embedded in polymers, as evidenced in modern biology. The apparent importance of genetics has underlined much of the allure of the RNA-First model, forcing this biological notion onto prebiotic chemistry. As argued above, the emergence of RNA chemistry and its incorporation into a protogenome, a conceivable precursor to the RNA-World, must have occurred as a result of a long evolutionary process through the organization and specialization of protobiological molecular networks [22,46]. This gradual transformation constitutes the next investigative avenue for RNA-World supporters, mapping the mechanistic principles underlying the emergence of stable genetic material from autocatalytic molecular systems.

Finally, a common defense for the RNA-First hypothesis is that despite its probabilistic hurdles, it remains the only available framework capable of explaining both the origin of life and the advent of genetics. This represents a false dichotomy. By prioritizing research into RNA-dominant systems, we risk ignoring the majority of chemical space and the fundamental dynamics necessary to initiate evolution from such prebiotic clutter. Collective autocatalytic sets represent a more statistically plausible entry point into evolution within messy abiotic environments. Persisting with a problematic hypothesis for the sake of conceptual convenience, rather than exploring the emergent properties of chemically diverse networks, potentially obscures the very mechanisms that facilitated the eventual recruitment of RNA and its subsequent specialization into its biological role.

## 4. Conclusions

The persistence of the RNA-First paradigm is driven by a powerful confluence of factors: the strong biochemical evidence of RNA’s deep ancestry, the foundation of analytical data for biomolecules compared to alternative abiotic chemistries, and a historical preference for sequence-centric informational models. However, this essay argues that the possibility that RNA served as life’s primal substrate is fundamentally implausible, and its pursuit projects biochemical biases onto abiotic chaos, often to bypass the daunting complexity of protobiology. RNA-First research has mainly promoted selective evidence of possibilities without demonstrating accurate prebiotic probabilities. It is far more plausible that a preceding protocellular evolutionary stage, comprising chemically diverse, catalytically active molecular networks, facilitated the eventual emergence of RNA and its central genetic role. Research into life’s origin must embrace the immense combinatorial space of abiotic chemistry and shift its focus toward the principles of emergent, evolving molecular systems. Only by understanding these pre-genetic networks can we explain how life transitioned from abiotic chemical messiness to the specialized innovation of the RNA-World.

## Data Availability

No new data were created or analyzed in this study. Data sharing is not applicable to this article.
